# EGCG and Taxifolin Modulate Secretory Activity and Expression of Dentinogenesis Markers in Odontoblast-Like Cells

**DOI:** 10.1155/sci5/3233536

**Published:** 2025-02-28

**Authors:** Cristiane Duque, Rafaela Laruzo Rabelo, Gabriela Pacheco de Almeida Braga, Igor Paulino Mendes Soares, Maria Eduarda de Souza, Daniela Alvim Chrisostomo, Josimeri Hebling, Carlos Alberto de Souza Costa, Anuradha Prakki, Luís Octavio Regasini

**Affiliations:** ^1^Universidade Católica Portuguesa (UCP), Faculty of Dental Medicine, Center for Interdisciplinary Research in Health (CIIS), Estrada da Circunvalação, s/n 3504-505, Viseu, Portugal; ^2^São Paulo State University (UNESP), Department of Preventive and Restorative Dentistry, Araçatuba Dental School, R. José Bonifácio, 1193, Araçatuba 16015-050, Brazil; ^3^São Paulo State University (UNESP), Department of Dental Materials and Prosthodontics, Araraquara School of Dentistry, R. Humaitá, 1680, Araraquara 14801-385, Brazil; ^4^University of Toronto, Department of Clinical Sciences-Restorative Dentistry, Faculty of Dentistry, 124 Edward St, Toronto, Ontario M5G 1G6, Canada; ^5^São Paulo State University (UNESP), Department of Morphology, Genetics, Orthodontics and Pediatric Dentistry, Araraquara School of Dentistry, R. Humaitá, 1680, Araraquara 14801-385, Brazil; ^6^São Paulo State University (UNESP), Department of Physiology and Pathology, Araraquara School of Dentistry, R. Humaitá, 1680, Araraquara 14801-385, Brazil; ^7^São Paulo State University (UNESP), Department of Chemistry and Environmental Sciences, Institute of Biosciences, Humanities, and Exact Sciences, R. Cristóvão Colombo, 2265, 15054-000, São José do Rio Preto, Brazil

**Keywords:** alkaline phosphatase, cytotoxicity, dentin mineralization, flavonoids, gene expression

## Abstract

Odontoblasts are cells specialized in dentin matrix deposition and the first line of defense when the dentin–pulp complex is injured by pathological processes, such as dental caries and trauma. Natural compounds, such as flavonoids, could be useful to stimulate odontoblast activity and reparative dentinogenesis in vital pulp therapies, especially in immature permanent teeth. This study evaluated the effect of flavonoids on odontoblast secretory activity and the expression of dentinogenesis markers. The effect of flavonoids was evaluated on phenotypic mineralization markers (alkaline phosphatase (ALP) activity and mineralized nodule deposition) by colorimetric assays and on the expression of *Alpl*, *Mmp2*, *Mmp9*, *Dmp1*, and *Dspp* genes in odontoblast-like cells by quantitative polymerase chain reaction. Most of the flavonoids did not show toxicity between 100 and 25 μM. In distinct concentrations, epigallocatechin gallate (EGCG), taxifolin, myricetin, quercetin, and kaempferol stimulated the activity of ALP and increased mineralized nodule deposition. However, the highest effect on those phenotypic markers was observed after EGCG and taxifolin treatments. Then, they were selected for evaluation of gene expression. mRNA levels of *Dmp1* and *Dspp* highly increased with taxifolin treatment, and *Alpl* expression was increased for both taxifolin and EGCG groups, without difference between them. *Mmp2* and *Mmp9* expression was not affected by these flavonoids. In conclusion, EGCG and taxifolin positively affect phenotypic mineralization markers; in particular, taxifolin highly stimulates early- and late-stage dentinogenesis genes.

## 1. Introduction

Dental caries or trauma can produce injuries to pulp tissues; however, depending on the extension of the pulp exposure and clinical status, the time between the trauma and treatment, and the degree of root formation, appropriate vital pulp therapy (VPT) can be successfully employed for the recovery of the dentin–pulp complex [1]. VPT, also known as direct pulp capping or pulpotomy, comprises the treatment of remaining pulp with an agent able to promote pulp healing and induce dentin repair [1]. Apexogenesis, or physiologic root end development and formation, can be achieved in immature permanent teeth with reversible pulpitis in the absence of periapical pathologies by VPT [1, 2]. Histological analysis of traumatized pulp shows that in teeth with crown fractures, the extension of inflammatory reaction does not reach 2 mm from the exposed surface and if these injured tissues are removed within 48 h, cells from noninflamed radicular pulp can reorganize and continue root development [2, 3].

Odontoblasts are cells specialized in dentin matrix deposition, which occurs during odontogenesis, throughout the life of the tooth, and when a tooth is injured by pathological processes. They are the first line of defense of the pulp–dentin complex and consequently the first ones to suffer deleterious effects [4]. The dental pulp is connected to the apical papilla in the apical portion of the tooth in immature teeth, and cells from both tissues are important for root formation [5]. The dentinogenesis process involves a wide range of enzymes and collagenous and noncollagenous proteins (NCPs). Alkaline phosphatase (ALP) is an enzyme present on the external surface of matrix vesicles (MVs) produced by odontoblasts, responsible for the hydrolysis of extracellular inorganic pyrophosphate in inorganic phosphate to form hydroxyapatite crystals and promoting dentin mineralization. Therefore, ALP activity can be used as an early-stage marker of mineralization [6, 7]. Metalloproteinases (MMPs) are also found in MV in dentin. They are a family of Zn2+- and Ca2+-dependent enzymes, which participate in the organization of extracellular matrix (ECM) and compartments during dentin formation. MMP-2 and MMP-9 (gelatinases) are secreted by odontoblasts and activate TGF-ß either in mineralized dentin or at the dentin–pulp border and then produce complex effects on the defense of the dentin–pulp complex [8]. The NCPs, dentin matrix protein 1 (DMP1) and dentin sialophosphoprotein (DSPP), are members of the family of small integrin-binding ligand N-linked glycoproteins (SIBLINGs), and they are commonly detected in ECM and play an important role in the late stage of dentin mineralization [6].

For several years, calcium hydroxide (CH) has been used in VPT and is considered a gold standard for its capacity to induce reparative dentin in teeth with exposed pulp. However, studies have reported inflammation and necrosis of the pulp surface, the presence of defects in dentin bridges, pulp chamber obliteration, and material degradation over time after VPT with CH [9]. Considering the limitations of CH, alternative choices of bioagents are interesting to provide more significant induction of dentin formation and higher biocompatibility.

Flavonoids are an important class of natural products usually found in the human diet with a broad spectrum of health-promoting effects, including antioxidative, anti-inflammatory, antimicrobial, and anticarcinogenic properties [10, 11]. Besides these properties, the stimulatory effects of flavonoids, such as quercetin, myricetin, epigallocatechin gallate (EGCG), taxifolin, chrysin, and pinocembrin, on osteoblast differentiation, ALP activity, collagen deposition, and bone mineralization were also reported in several studies [12–16]. In addition, taxifolin and EGCG significantly inhibited apoptosis and inflammation and increased the proliferation and differentiation of dental pulp cells [17, 18]. EGCG also promoted the differentiation of human dental pulp stem cells in odontoblast-like cells and increased mineral deposition in osteogenic media [19]. EGCG and taxifolin also increased mineralization markers' expression in stem cells from the apical papilla, in the presence of mineralizing agents [20]. Although previous studies have shown positive effects of some flavonoids on osteogenic and/or odontoblastic cells, no study was found comparing their stimulatory effects on odontoblasts and dentin mineralization. The aim of this study was to evaluate the cytotoxicity and effect of flavonoids on phenotypic and genotypic mineralization markers in odontoblast-like cells and evaluate if there is a structure–activity relationship. Only the flavonoids with the highest effect on phenotypic markers were selected for evaluation of gene expression. The null hypothesis tested is there was no difference among the flavonoids considering cell viability and the dentinogenesis markers evaluated.

## 2. Materials and Methods

The following flavonoids were evaluated: (−)-EGCG (#E4143), taxifolin (#78666), myricetin (#70050), quercetin (#Q4951), chrysin (#C80105), kaempferol (#K0133), and pinocembrin (#P5239). The compounds were all solids, of analytical standard, and > 90% (TLC). All compounds were filtered on a 0.22-μm membrane filter. Flavonoid stock solutions were prepared and frozen in dimethylsulfoxide (DMSO). For the experiments, DMSO concentration in flavonoid solutions was below 0.5%, which is not cytotoxic and does not have an effect on cell markers [16, 20]. The compounds and reagents were purchased from Sigma-Aldrich (St. Louis, MO, USA). All experiments were performed in triplicate on three independent days (*n* = 9), except by gene expression (two independent days, *n* = 6).

### 2.1. Viability of Odontoblast-Like Cells

The immortalized odontoblast-like cells (mouse dental papilla cells (MDPC-23)) [21] were kindly provided by Dr. Hanks and Dr. Nor from the University of Michigan and cultivated in DMEM medium supplemented with 10% fetal bovine serum (FBS), the antibiotics penicillin (100 IU/mL) and streptomycin (100 μg/mL), and 2 mmol/L glutamine and incubated in an incubator containing 5% CO_2_ at 37°C, being subcultured every 2 days [16, 22]. Cells were seeded (1.5 × 10^5^ cells/well) in 96-well microplates and preincubated for 24 h. After this period, the culture medium was aspirated and the cells were subjected to treatment with the flavonoids EGCG, taxifolin, myricetin, quercetin, chrysin, kaempferol, and pinocembrin diluted in DMEM medium in serial concentrations of 100 to 25 μM. Cell viability was evaluated by the resazurin method (Sigma-Aldrich) after 24 h and 48 h of treatment. The concentrations used for the flavonoids were based on previous studies [16, 20]. At each time, the culture medium was aspirated, and the cells were incubated with resazurin (70  μM) in the culture medium and kept for 4 h in an incubator. The absorbance was then read at 570 nm and 600 nm in a spectrophotometer (Synergy H1, BioTek, Winooski, VT, USA). The values were converted into a percentage of cell viability considering the growth in DMEM as 100% and the means determined for each group.

### 2.2. ALP Activity Assays

Cells were seeded in 48-well microplates (1.5 × 10^5^ cells/well) and preincubated for 48 h. After that, flavonoids were inserted daily in DMEM medium (at 100, 50, and 25 μM) until completing 48 h, followed by a daily exchange of osteogenic medium (DMEM containing 10% FBS, antibiotics, and glutamine supplemented with 10 nmol/L of β-glycerophosphate, 50 μg/mL of ascorbic acid, and 1.8 mmol/L KH_2_PO_4_) until completing 8 days [16, 20, 22]. The ALP assays were performed following the instructions of the ALP kit manufacturer (Labtest Diagnostics S.A., Lagoa Santa, MG, Brazil), and absorbance was measured at 590 nm with a spectrophotometer (Synergy H1). ALP activity was calculated from a standard curve using known enzyme concentrations [16, 23]. The final ALP data in percentages (in relation to the osteogenic DMEM control—100%) were normalized by the values obtained for cell viability in percentages (in relation to the DMEM control—100%), determined by the resazurin method performed parallelly on day 8.

### 2.3. Alizarin Red Staining and Quantification of Mineralized Nodules

The tests were conducted as described in the previous item, but until completing 13 days of daily exchange of osteogenic medium, and biomineralization was evaluated by staining with alizarin red S (Sigma-Aldrich) [20, 22]. Briefly, 40 mmol/L of alizarin red S was prepared in distilled water, adjusted to pH 4.2 with ammonia hydroxide, and then applied to the cells for 10 min at room temperature under gentle agitation. The cells were then washed with distilled water twice and allowed to dry at room temperature. Images of cell cultures stained with alizarin red S were obtained using an inverted microscope. After obtaining the images, 10% cetylpyridinium chloride in PBS was added to the wells and left under stirring for 15 min to dissolve the alizarin red S salts. Three 100 μL aliquots from each well were transferred to another microplate and evaluated in a spectrophotometer considering a wavelength of 562 nm. The results in absolute values were converted to % considering the osteogenic DMEM control as 100%.

### 2.4. Gene Expression of Dentinogenesis Markers by Quantitative PCR

Cell preincubation in microplates was conduced as described in item 2.2 and 2.3 (1.5 x 10^5^ cells/well for 48h). Afterward, cells were treated with EGCG and taxifolin at 50μM for 48h and the culture medium was changed by osteogenic DMEM until completing 13 days. After that, the total RNA was extracted, isolated, and purified using the TRIzol reagent (Invitrogen), strictly following the protocols recommended by the manufacturer. Then, DNase I treatment was performed (DNase I, Grade II from bovine pancreas, Sigma-Aldrich), and 500 ng RNA from each well was used for single-stranded cDNA synthesis (Superscript III Kit, Invitrogen, Thermo Scientific) using random hexamer primers according to the protocols provided by the manufacturer [16, 24]. qPCRs were performed using the StepOnePlus Real-Time PCR System (Applied Biosystems) from 2-μL cDNA templates, TaqMan Fast Advanced Master Mix (Applied Biosystems), and predesigned assays of primers and probes labeled with FAM reporter dye (Applied Biosystems) for *Alpl* (Rn01516028 m1), *Mmp2* (Rn01538170_m1), MMP-9 (Rn00579162_m1), *Dspp* (Rn02132391 s1), and *Dmp1* (Rn01450122 m1) sequences. The relative gene expression was calculated using 2^–ΔΔCq^ equations, with the *GAPDH* (Rn01516028 m1) expression used as the previously validated reference gene, and the negative control group (DMEM) for data normalization [25]. The RT-qPCR protocol was performed in two different runs, and the final number of replicates was *n* = 6.

### 2.5. Statistical Analysis

Data from cell viability, ALP activity, mineralized nodule formation, and gene expression are presented as mean ± SD and analyzed using ANOVA and Tukey test. Dunnett tests were used to compare the results obtained by flavonoids with those from untreated control. SPSS 17.0 software (SPSS Inc, Chicago IL, USA) was used to run the statistical analysis, considering *p* < 0.05.

## 3. Results

### 3.1. Viability of Odontoblast-Like Cells

**Figure 1:**
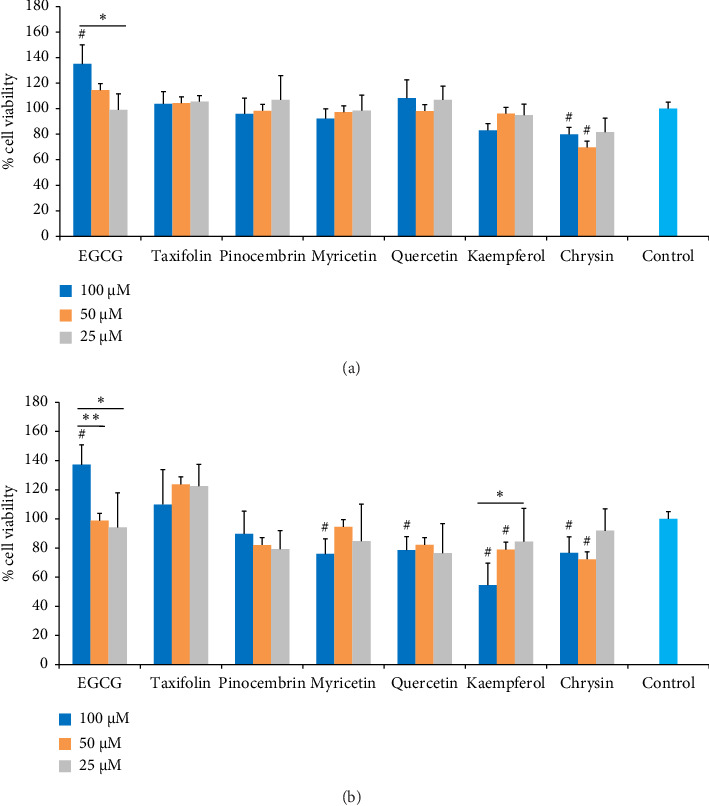
Effect of flavonoids on the viability of MDPC-23 odontoblast-like cells, after 24 h (a) and 48 h (b) of treatment followed by resazurin staining. Control = DMEM. Data represent the mean ± SD of triplicate in three independent experiments (*n* = 9). ⁣^∗^*p* < 0.05 and ⁣^∗∗^*p* < 0.01 (⁣^∗^ and ⁣^∗∗^significant difference among the flavonoids, according to ANOVA/Tukey); ⁣^#^*p* < 0.05 and ⁣^##^*p* < 0.01 (⁣^#^ and ⁣^##^significant difference compared to untreated control, according to Dunnett's tests).

Figure 1 shows the viability results of odontoblast-like cells (MDPC-23) after 24 and 48 h of exposure to flavonoids and untreated control. At 24 h (Figure 1(a)), cell viability was above 80% when exposed to all flavonoids, independent of the concentration, except for chrysin at 100 and 50 μM (79.9% and 69.6%, respectively) compared to the untreated control. At 48 h (Figure 1(b)), cell viability was above 70% for all flavonoids at all concentrations, except for kaempferol (54.6%) at 100 μM compared to the untreated control. Although myricetin and quercetin at 100 μM, chrysin at 100 and 50 μM, and kaempferol at 50 μM differed from the control, cell viability ranged from 72.3% to 79%. EGCG stimulated cell metabolism by 135% at 24 h and 137% at 48 h, differing from the other flavonoids, at 100 μM.

### 3.2. ALP Activity

**Figure 2:**
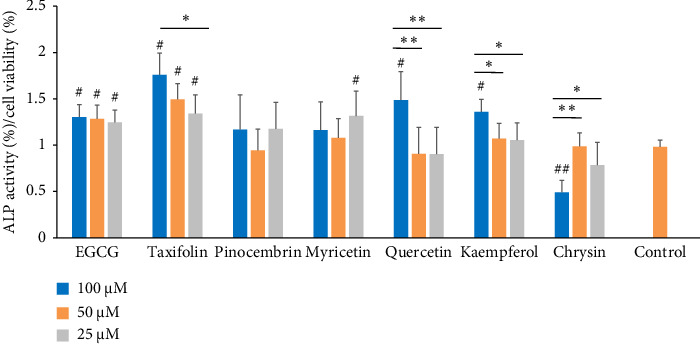
Alkaline phosphatase activity after 48 h of exposure to flavonoids and 8 days of growth in an osteogenic medium. Control = osteogenic DMEM. Data represent the mean ± SD of triplicate in three independent experiments (*n* = 9). ⁣^∗^*p* < 0.05; ⁣^∗∗^*p* < 0.01 (⁣^∗^ and ⁣^∗∗^the significant difference among the flavonoids, according to ANOVA/Tukey); ⁣^#^*p* < 0.05; ⁣^##^*p* < 0.01 (⁣^#^ and ⁣^##^the significant difference compared to control, according to Dunnett's tests).

Figure 2 shows ALP activity obtained by MDPC-23 cells after exposure to flavonoids. EGCG and taxifolin (from 100 to 25 μM), myricetin (25 μM), and quercetin and kaempferol (both at 100 μM) stimulated ALP activity, differing statistically from the untreated control. Taxifolin, quercetin, kaempferol, and EGCG, all at 100 μM, stimulated the highest effect on ALP, increasing its activity by 1.75-, 1.48-, 1.35- and 1.32-fold compared to the control. At 50 μM, ALP activity was statistically higher for cells treated only with taxifolin and EGCG compared to the control. Only chrysin at 100 μM reduced ALP activity, compared to the untreated control.

### 3.3. Deposition of Mineralized Nodules

**Figure 3:**
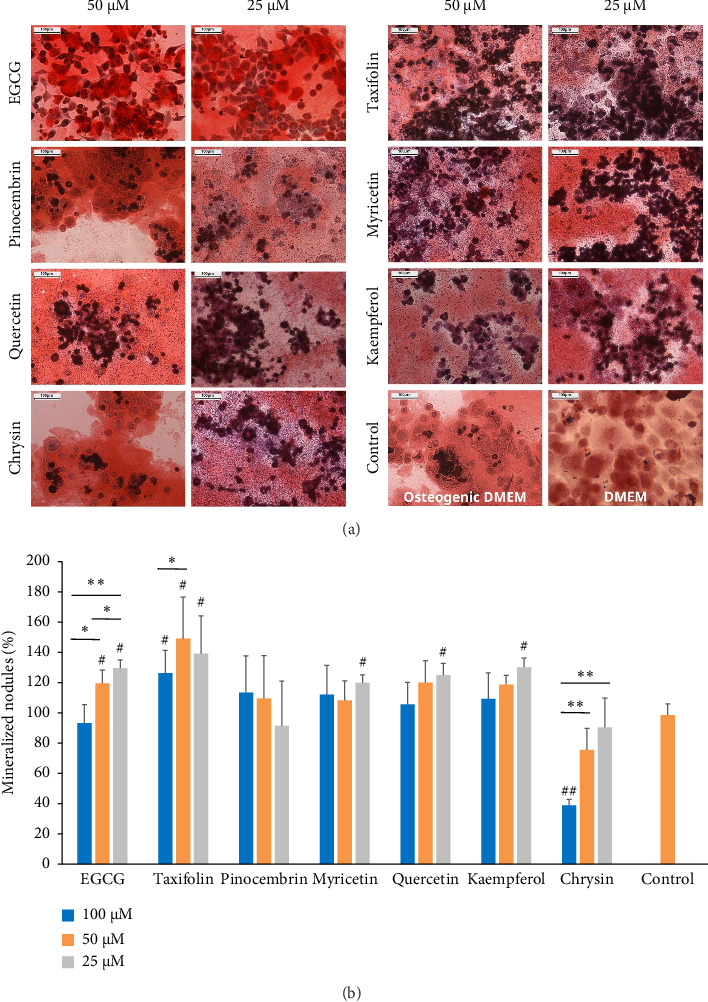
(a) Representative images of alizarin red staining showing the mineralization ability of MDPC-23 cells after 48 h of treatment with EGCG, taxifolin, pinocembrin, myricetin, quercetin, kaempferol, and chrysin at 50 μM and 25 μM and 13 days of growth, compared to osteogenic DMEM. (b) Production of mineralized nodules (% in relation to untreated control) after treatment with flavonoids and growth in osteogenic medium. Control = osteogenic DMEM. Data represent the mean ± SD of triplicate in three independent experiments (*n* = 9). ⁣^∗^*p* < 0.05; ⁣^∗∗^*p* < 0.01 (⁣^∗^ and ⁣^∗∗^the significant difference among the flavonoids, according to ANOVA/Tukey); ⁣^#^*p* < 0.05; ⁣^##^*p* < 0.01 (⁣^#^ and ⁣^##^the significant difference compared to control, according to Dunnett's tests). Scale bars represent 100 μm.

Figure 3(a) shows representative images of alizarin red staining demonstrating the mineralization ability of MDPC-23 cells after treatment with EGCG, taxifolin, pinocembrin, myricetin, quercetin, kaempferol, and chrysin at 50 μM and 25 μM, compared to osteogenic DMEM and DMEM. The highest deposition of mineralized nodules was observed after treatments with EGCG and taxifolin at 50 and 25 μM compared to the other groups and controls—osteogenic DMEM and control nonosteogenic DMEM. The data relating to the percentage of mineralized nodules deposition by MDPC-23 cells after exposure to flavonoids are shown in Figure 3(b). EGCG and taxifolin (at 50 μM) stimulated nodule deposition up to 120% and 148%, respectively, differing statistically from the control with no treatment. EGCG, taxifolin, myricetin, quercetin, and kaempferol (at 25 μM) stimulated deposition up to 129%, 139%, 119%, 124%, and 130%, respectively, compared to the control.

### 3.4. Gene Expression of *Alpl*, *Mmp2*, *Mmp9*, *Dmp1*, and *Dspp*

**Figure 4:**
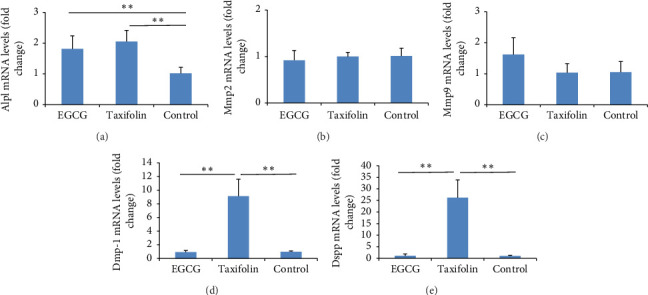
Expression of alkaline phosphatase (*Alpl*) (a), metalloproteinase-2 (*Mmp2*) (b) and (*Mmp9*) (c), dentin matrix protein-1 (*Dmp1*) (d), and dentin sialophosphoprotein (*Dspp*) (e) from MDPC-23 cells after 48 h of exposure to EGCG and taxifolin at 50 μM and 13 days of growth. Control = osteogenic DMEM. Data represent the mean ± SD of triplicate in two independent experiments (*n* = 6). ⁣^∗^*p* < 0.05; ⁣^∗∗^*p* < 0.01 (⁣^∗^ and ⁣^∗∗^the significant difference among the flavonoids, according to ANOVA/Tukey).

Considering the highest effect of EGCG and taxifolin on ALP activity and deposition of mineralized nodules, only these flavonoids at 50 μM were chosen for the evaluation of gene expression of dentinogenesis markers.  Figure 4 shows mRNA levels of *Alpl*, *Mmp2*, *Mmp9*, *Dmp1,* and *Dspp* when odontoblast-like cells were treated with EGCG or taxifolin at 50 μM for 48 h and grown for 13 days. *Alpl* expression was increased for EGCG and taxifolin, without difference between them (1.81 and 2.05 times, respectively). None of the flavonoids affected *Mmp2* and *Mmp9* expressions. Gene expression of *Dmp1* and *Dspp* highly increased with taxifolin treatment, 9.13 and 26.19 times, respectively.

## 4. Discussion

In the current study, the flavonoids EGCG, taxifolin, myricetin, quercetin, and kaempferol stimulated ALP activity and mineral deposition, at distinct concentrations, in odontoblast-like cells. However, EGCG and taxifolin had the highest effect on ALP activity and mineralized nodules deposition, as well as increased gene expression of dentinogenesis markers compared to the osteogenic control. Then, the proposed null hypothesis was rejected.

The effect of flavonoids on pulp cells has been studied in odontoblast-like cells and human dental pulp stem cells, with effects observed on stimulation of differentiation, matrix secretion, and mineral deposition [17–20, 26–31]. However, this is the first study comparing compounds representative of the five groups of flavonoids: flavone (chrysin), flavonol (quercetin, kaempferol, and myricetin), flavanone (pinocembrin), flavanonol (taxifolin), and flavanol (EGCG), considering their previous effect on osteogenic/odontoblastic cells. Among the flavonoids, taxifolin and EGCG were the most effective compounds in stimulating the expression of phenotypic and genotypic mineralization markers by odontoblast-like cells. We could not find a direct structure–activity relationship between EGCG and taxifolin since they have distinct chemical structures and belong to different groups of flavonoids. However, both compounds are characterized by the presence of two chiral centers at C2 and C3, resulting in four possible stereoisomers, with the (2R, 3R) isomer being the most prevalent in nature and chosen for the study. Another chemical characteristic in common is the presence of five hydroxyl groups (OH) in their structure backbone. The number and position of OH along with conjugation and resonance effects have been associated with the biological activities of flavonoids, including scavenging free radicals and chelating metal ions [32].

The effect of EGCG and taxifolin on mineralization markers has been more widely studied in osteoblasts or pluripotent stem cells [16–20, 26–28]. In the present study, EGCG and taxifolin did not affect odontoblast viability from 25 to 100 μM. They increased ALP, mineral deposition, and expression of genes associated with dentin mineralization, at 50 μM. In the literature, EGCG did not affect the viability of odontoblast-like cells at concentrations between 2.5 and 160 μM and modulated the secretion of several pro- and anti-inflammatory cytokines [33, 34]. ALP and mineral deposition were stimulated by the presence of EGCG and taxifolin, at 10–12.5 μM, in dental papilla stem cells (SCAP) after induction of proliferation/migration [20, 26]. Odontogenic differentiation was observed by the increased expression of mineralization markers such as *DMP1* and *DSPP* by SCAP in the presence of EGCG [26]. Differently, EGCG at 10 μg/mL (21.8 μM) did not have an effect on proliferation and calcium nodule formation by hDPSC but inhibited the inflammatory response and apoptosis caused by hypoxia injury [17].

To the best of our knowledge, the potential of taxifolin to induce mineralization markers in pulp cells has not yet been described. In the present study, besides the positive effect on ALP activity and nodule deposition, taxifolin highly increased *Dmp1* and *Dspp* expression, two important markers of dentin mineralization, as well as stimulating ALP expression with no difference when compared to EGCG. Taxifolin at 10 μM significantly reduced the apoptosis of hDPSC under inflammation and hypoxia conditions [18]. Treatment with taxifolin increased the osteogenic differentiation of human bone marrow mesenchymal stem cells (hBMSCs) without showing cytotoxicity, in addition to stimulating the activity of ALP at concentrations of 50 and 100 μM and the production of mineralized nodules [28], as observed in the present study with odontoblast-like cells. A recent study observed that pretreatments with taxifolin for 24 h and 72 h at 10 μM increased ALP activity, mineralized nodule deposition, and ALP and COL-1 gene expression by osteoblastic Saos-2 cells [16].

Compared to taxifolin and EGCG, the other flavonoids tested had no effect or lower or negative effects on mineralization markers. Myricetin at 25 μM and quercetin and kaempferol at 100 μM increased ALP, pinocembrin did not differ from the control, and chrysin, especially at 100 μM, was cytotoxic and had negative effects on mineralization markers. Previous studies with those flavonoids have shown different results compared to the current study [29–31]. Myricetin promoted proliferation up to 72 h and the expression of the key marker of odontogenic biomineralization and differentiation, such as *DMP1*, in an odontoblast-like cell model at concentrations between 150 and 600 μM, in addition to causing MMP inhibition without affecting the mechanical properties of dentin [29]. In hDPSC, quercetin at 2.5 μM also increased ALP activity, *DSPP* expression, and mineral deposition [30]. Kaempferol at 0.001 μM promoted the proliferation, ALP activity, and mineral deposition of periodontal ligament stem cells [30]. MG-63 human osteoblasts were treated with quercetin and kaempferol at 50 μM, and both compounds increased ALP activity 1.5 and 1.7 times when compared to the control, in a time- and dose-dependent manner [31].

Pinocembrin isolated from the leaves of *Alpinia zerumbet* at 150 μM showed enhanced ALP activity and biomineralization and increased mRNA expression of osteoblast-related genes *ALP* and osteocalcin in MC3 T3-E1 osteoblastic cells [15]. Different from the present study, in another investigation, chrysin was not cytotoxic and induced phenotypic mineralization markers, such as ALP and mineralized nodules [31]. In addition, this flavonoid also upregulated the expression of osteogenic proteins and induced osteogenic differentiation of hDPSC [31]. However, the concentrations of chrysin (0.001–20 μM) and time of treatment (5 days) used in this previous study were different from those chosen for the present study [31]. Overall, differences in the methodologies, especially related to the compound's concentration, time of treatment, and types of cell lines, can explain the divergent results between the literature and the present study.

In the current study, EGCG and taxifolin had no effect on *Mmp2* and *Mmp9* expression. MMPs are members of an enzyme family depending on zinc ions for catalytic activity, responsible for regulating the normal turnover of ECM macromolecules such as collagens, proteoglycans, and fibronectin. MMP-2 and MMP-9 seem to have an important role in the pathogenesis of pulp and periapical destruction because they degrade gelatin and type IV collagen, the major constituents of basement membranes [8, 35]. The flavone baicalein at low concentrations effectively inhibited the expression of *MMP2*, *MMP9*, cathepsin-B, and cathepsin-K in human pulp cells and improved the strength of aged resin-dentin bonding [36]. Baicalein also attenuated inflammatory factors such as interleukin-1β and tumor necrosis factor-α, as well as MMP-2 at both mRNA and protein levels. Furthermore, this flavonoid stimulated osteogenic differentiation and increased ALP and nodule deposition, as well as the expression of osteogenic markers in periodontal ligament cells treated with lipopolysaccharides [37]. EGCG in gelatin sponges was inserted into bone defects in rats where it decreased *MMP2* and *MMP9* expression and increased bone formation [38]. No studies were found evaluating the effects of taxifolin on *MMP2* and *MMP9* expression in pulp cells or even in osteoblasts. It is important to point out that qPCR provides insights into gene expression changes, but it does not directly measure functional consequences. Post-transcriptional processes, including splicing, RNA modifications, and translation efficiency, can significantly influence protein expression. This study could be complemented with immunoassays, such as western blot and ELISA, for protein quantification of biomarkers.

The effect of taxifolin and EGCG on mineralization markers in odontoblast-like cells was comparable to CH, a gold-standard compound used in endodontics, at the same concentrations [39]. CH, at 50 and 25 μM, was cytocompatible and increased ALP and mineralized nodules deposition by MDPC-23 cells [39]. The results also were similar to ampelopsin (or dihydromyricetin), a flavanonol with chemical structure analog to taxifolin, differing by the presence of one more hydroxyl group linked to phenolic ring B [39]. In clinical practice, CH is used for VPT in a concentration (≥ 1 mg/mL or ≥ 13 mM) much higher than those mentioned before. However, even diluted (at 0.06 mg/mL or 0.8 mM), CH reduced the metabolic activity and cell proliferation and promoted apoptosis or necrosis in the culture of MDPC-23 cells [40].

Overlapping mechanisms of action of flavonoids on mineralization are likely to occur. Previous proteomic results with dentin extracts inferred a selective EGCG binding mechanism with NCPs [23]. Moreover, molecules containing phenyl hydroxy groups are known to act as ligands to bind to calcium and therefore to have the ability to enhance mineral deposition [24]. Although the studies were focused on osteoblastic cells, there is evidence that activation of calcium channels influences cellular signal transport [40], bone resorption [41], and cell proliferation [42]. Saponara et al. [43] evaluated several flavonoids, and 24 of them were activators or deactivators of calcium channels, measured in myocytes from the rat tail artery. A recent study evaluating the structure–activity relationship validated the bone formation capacity of flavonoids through their high activating effect on the voltage-controlled calcium channel for osteogenesis [44]. More studies are necessary to understand which mechanisms are activated by flavonoids to increase the biomineralization ability of pulp cells.

It is crucial to add that flavonoids are particularly promising in the context of dentistry, considering their multifunctional properties and the etiology/development of dental infections. Particularly in endodontics, bacterial contamination and severe pulp inflammation can occur when teeth are affected by dental caries or trauma. Then, previously reported antimicrobial and anti-inflammatory properties of flavonoids could reduce or even eliminate bacteria from root canals and control inflammatory response [45–48]. In addition, as explored in the present study, they could induce dentin repair, without causing toxicity to remaining cells. In immature permanent teeth, flavonoids could be an alternative to conventional treatments, promoting pulp healing and allowing physiologic root end formation. However, for clinical application, they could be incorporated in drug carriers, such as injectable natural or synthetic polymers, to promote the controlled release of compounds, extending their biological effects and reducing the frequency of intracanal medication changes and consequently interappointment visits [49].

## 5. Conclusions

In conclusion, EGCG and taxifolin have positive effects on mineralization markers, and, particularly, taxifolin highly stimulated early and late genetic markers of dentinogenesis.

## Data Availability

The data that support the findings of this study are partially available in the Institutional Repository UNESP, at http://hdl.handle.net/11449/214906, reference number [48], and also available from the corresponding author upon reasonable request.
